# COVID-19 incidence on Emergency Departments accesses. Health need and fear of infection, what wins?

**DOI:** 10.1093/eurpub/ckac129.541

**Published:** 2022-10-25

**Authors:** C Quercioli, R Bosco, G Bova, M Mandò, S Dei, R Gusinu, G Messina

**Affiliations:** 1 Post Graduate School of Public Health, University of Siena, Siena, Italy; 2 Hospital Healthcare Management, Poggibonsi Hospital, Local Health Unit Toscana Sud-Est, Poggibonsi, Italy; 3 University Hospital “Santa Maria alle Scotte”, Siena, Italy; 4 Emergency Department Director, Local Health Unit Toscana Sud-Est, Arezzo, Italy; 5 Local Health Unit Toscana Sud-Est, Arezzo, Italy; 6 University Hospital “Santa Maria alle Scotte”, Siena, Italy; 7 Department of Molecular and Developmental Medicine, University of Siena, Siena, Italy

## Abstract

**Introduction:**

The COVID-19 pandemic has changed the patterns of access to the Emergency Department (ED), but it is unclear whether this change was due to COVID-19 incidence or the lockdown imposed by law.

**Aim:**

To evaluate the association between trends of ED accesses and COVID-19 incidence in the period 1 January - 31 December 2020.

**Material and methods:**

The data of accesses to the ED per month and severity triage code of 14 hospitals in the Southeast Tuscany (Italy, Provinces of Siena, Arezzo, Grosseto) were obtained from hospitals data warehouses. Data on new cases of COVID-19 infection (obtained by the Ministry of Health) for the 3 provinces were used to calculate the incidence of infection. Hospitals were classified in 4 categories based on beds number, medical specialties offered, services provided. Differences in ED accesses by month, triage code and hospital type were investigated by a Kruskal-Wallis analysis of variance. Association between ED accesses and COVID-19 incidence was evaluated using a random effect panel data analysis adjusting for hospital type and triage code.

**Results:**

A total of 268,072 ED accesses have been studied. Their trends saw a strong decrease in correspondence of the first pandemic peak, subsequently they are increased and then decreased again until the minimum peak in November 2020. COVID-19 incidence appeared to overlap, but in the reverse direction, with ED admissions trends. Monthly differences of the ED accesses were significant (p < 0.01) except for most severity code. There is a statistically significant inverse association between ED accesses and COVID-19 incidence (Coef. = -0.074, p < 0.001) except for most severe cases (triage code 1: Coef. = -0.028, p = 0.154).

**Conclusions:**

ED admissions trends followed the COVID-19 incidence independently from the period of lockdown except for the most severe cases. The fear to contract the infection seemed to discourage patients to access ED for diseases that were perceived as not serious.

**Key messages:**

• The pandemic has changed the lifestyle of people worldwide, modifying even the perception that the patient has of own state of health and their access to Emergency Department.

• The decrease in accesses involved less severe cases. Reflect on both the adequacy of accesses in the pre-pandemic period and on what is the best setting to manage these cases in the pandemic period.

